# Ulcerative Colitis Flare Post Isotretinoin Use: A Case Report

**DOI:** 10.7759/cureus.46429

**Published:** 2023-10-03

**Authors:** Maha AlHussein, Abdulaziz H AlHussain

**Affiliations:** 1 College of Medicine, Princess Nourah Bint Abdulrahman University, Riyadh, SAU; 2 Department of Dermatology, King Abdullah Bin Abdulaziz University Hospital, Riyadh, SAU

**Keywords:** general dermatology, flare-up, ulcerative colitis (uc), acne vulgaris, isotretinoin

## Abstract

Ulcerative colitis (UC) is an inflammatory bowel disease characterized by chronic intestinal inflammation. We present a case of UC flare induced by isotretinoin, a medication commonly used for the treatment of severe acne. A 36-year-old male with a history of UC in remission presented with moderate-severe cystic acne and was started on isotretinoin. After four months of treatment, he developed symptoms of UC flare, including bloody diarrhea. A colonoscopy revealed ulcerative pancolitis, and isotretinoin was discontinued. The patient was subsequently treated with adalimumab and azathioprine, achieving good disease control. This case highlights the potential link between isotretinoin and UC exacerbation, suggesting the need for further research to understand the underlying mechanisms and assess the risk associated with isotretinoin use in patients with UC.

## Introduction

Ulcerative colitis (UC) is a chronic idiopathic intestinal inflammatory disease that presents as abdominal pain, diarrhea, and hematochezia [1]. It is considered a part of the inflammatory bowel disease together with Crohn’s disease [1]. The exact cause of ulcerative colitis is yet to be determined, but various mechanisms have been proposed; these include genetic susceptibility and immune dysregulation [2]. Here, we report a case of ulcerative colitis flare induced by isotretinoin.

## Case presentation

A 36-year-old male with a history of left-sided ulcerative colitis since eight years ago on mesalazine oral and suppository, which had been in remission for over two years, presented to our dermatology clinic complaining of a 20-year history of moderate-severe cystic acne. The patient was previously on topical treatments with little improvement.

Our patient was started on 20 mg of isotretinoin capsule (Roaccutane) once daily after a gastroenterology consultation as well as a laboratory workup encompassing a liver function test, lipid profile, and complete blood count that were within the normal range.

After four months of oral isotretinoin, he developed loose bowel motions (>7/day), with blood that was yellow in color and non-watery, no abdominal pain, and no nausea or vomiting.

Investigations were done to rule out the differentials, including a complete blood count. Laboratory findings are summarized in Table 1.

 

**Table 1:** The patient's laboratory findings

Test	Result	Reference ranges	
White blood cells	6.70	4.00-11.00	10^9/L
Neutrophils	3.81	2.00-7.50	10^9/ L
Lymphocytes	1.65	1.00-4.40	10^9/ L
Eosinophils	0.13	0.10-0.70	10^9/L
Red blood cells	5.46	4.50-6.10	10^12/ L
Hemoglobin	130	135 – 180	g/L

Furthermore, a septic workup, stool calprotectin, stool *Clostridioides difficile* toxin (CDT), stool chart, in-and-output chart, abdominal X-ray, and ultrasound (US) of the kidney urinary bladder (KUB) were unremarkable.

**Figure 1:**
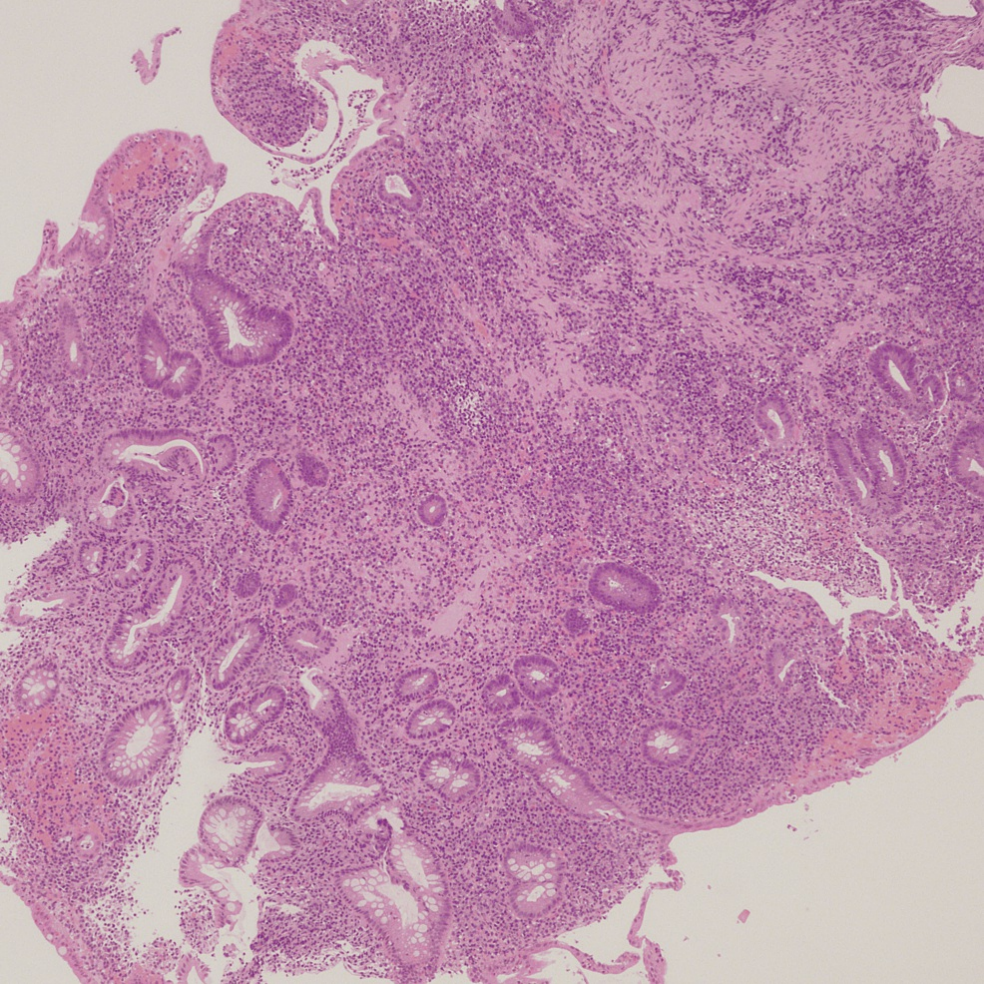
Ulcerative pancolitis with a Mayo score of 2-3; histology shows ulcerative colitis, extensive inflammation, crypt architectural distortion, crypt atrophy, and crypt loss

A colonoscopy revealed ulcerative pancolitis with a Mayo score of 2-3. Histology showed ulcerative colitis, extensive inflammation, crypt architectural distortion, crypt atrophy, and crypt loss (Figure 1).

**Figure 2:**
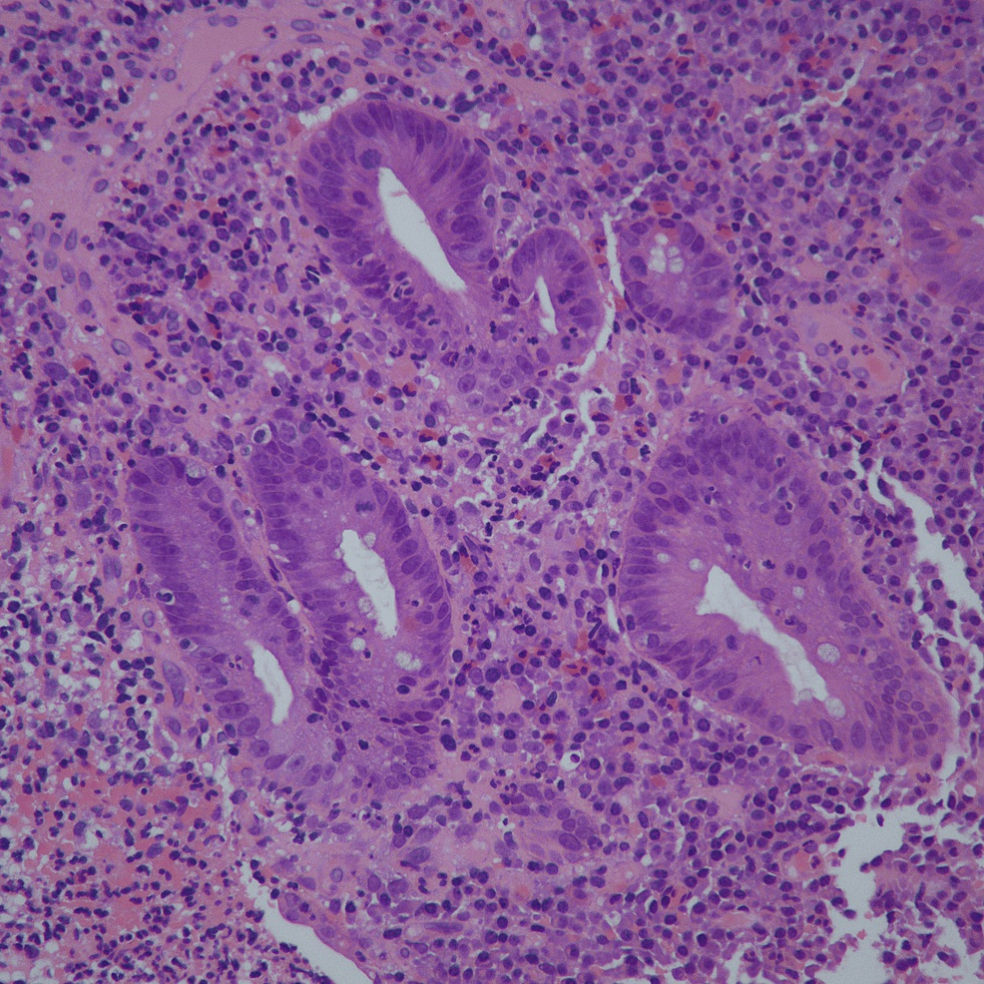
Cryptitis and eosinophilic infiltration in the lamina propria

Figure 2 shows cryptitis and eosinophilic infiltration in the lamina propria.

Since he developed the UC flare pancolitis, isotretinoin was discontinued. He was started on adalimumab (Humira) induction dose, then 40 mg subcutaneously (SC) once weekly, and azathioprine (50 mg per oral (PO) once daily) with good control.

## Discussion

Ulcerative colitis typically affects young adults, with an estimated prevalence of up to 30 million people by 2025 [3]. The clinical course of UC varies from a stable course with prolonged remission to recurrent flare-ups, which is the return of symptoms after a period of remission [1]. Reports have shown numerous factors contributing to UC relapses, such as genetic susceptibility and fecal incontinence [2]. Histologically, a distinct characteristic of UC is the identification of uninterrupted inflammation in the colon, exhibiting features such as redness, disrupted vascular pattern, bleeding, and ulcerations [4]. Here we report a UC flare following isotretinoin treatment, which is, to our best knowledge, the first case report of a UC exacerbation related to isotretinoin in Saudi Arabia.

Isotretinoin is a retinoid derivative of vitamin A that works by inhibiting sebaceous gland activity and has other anti-inflammatory and immunoregulatory properties [5]. Dermatologists consider isotretinoin to have an adequate safety profile; it is primarily used to treat resistant nodular acne [5]. Before using isotretinoin, patients should be aware of its most common adverse effects, including cheilitis and xerosis, and its teratogenicity [5]. Laboratory monitoring is indicated for patients on isotretinoin therapy due to potential abnormalities such as low high-density lipoproteins (HDLs) or elevated liver function tests [5].

There have been several case reports of isotretinoin-inducing or exacerbating ulcerative colitis. Bharmal et al. reported that a 27-year-old female with no medical history developed severe active colitis with multiple superficial ulcers after isotretinoin treatment lasting 16 weeks [6]. An additional case report reported that a 17-year-old boy was diagnosed with ulcerative colitis after an isotretinoin plan; the patient received a subtotal colectomy and ileostomy five months after the first presenting symptom [7]. In a case-control study conducted in 2010, it was found that Accutane is linked to a slight risk of developing ulcerative colitis [8]. The researchers, Crockett et al., suggested that higher dosages of Accutane might be associated with an increased risk of ulcerative colitis. [8]. According to another review article, isotretinoin has been reported to potentially initiate the onset of ulcerative colitis in individuals who are highly susceptible to the condition [9]. How retinoids cause or induce an intestinal inflammation flare is not yet fully comprehended. However, it is hypothesized that isotretinoin may impact T-cell function and adaptive immunity, although the exact mechanism is not fully understood. Additional research is required to validate the impact of isotretinoin on the progression of the disease. [9].

## Conclusions

In conclusion, this case report highlights the occurrence of an ulcerative colitis flare following treatment with isotretinoin, a medication primarily used for severe acne. While isotretinoin is generally considered safe and effective for its intended purpose, there have been reported cases of it inducing or exacerbating ulcerative colitis. This underscores the importance of considering potential adverse effects and monitoring patients closely during isotretinoin therapy. The exact mechanism by which isotretinoin may trigger an inflammatory bowel disease flare is still not fully understood, and further research is needed to explore this relationship. Healthcare providers should be aware of this potential association and exercise caution when prescribing isotretinoin to patients with a history of or susceptibility to ulcerative colitis. Overall, this case serves as a reminder of the complexity of drug-induced reactions and the need for individualized patient care in the context of inflammatory bowel diseases.
